# Imported Toxin-Producing Cutaneous Diphtheria — Minnesota, Washington, and New Mexico, 2015–2018

**DOI:** 10.15585/mmwr.mm6812a2

**Published:** 2019-03-29

**Authors:** Jayne Griffith, Catherine H. Bozio, Amy J. Poel, Kelly Fitzpatrick, Chas A. DeBolt, Pamela Cassiday, Cynthia Kenyon, Chad Smelser, Paula Snippes Vagnone, Karissa Culbreath, Anna M. Acosta

**Affiliations:** ^1^Minnesota Department of Health; ^2^Division of Bacterial Diseases, National Center for Immunization and Respiratory Diseases, CDC; ^3^Epidemic Intelligence Service, CDC; ^4^Washington State Department of Health; ^5^New Mexico Department of Health; ^6^Department of Pathology, University of New Mexico School of Medicine, Albuquerque, New Mexico.

From September 2015 to March 2018, CDC confirmed four cases of cutaneous diphtheria caused by toxin-producing *Corynebacterium diphtheriae* in patients from Minnesota (two), Washington (one), and New Mexico (one). All patients had recently returned to the United States after travel to countries where diphtheria is endemic. *C. diphtheriae* infection was not clinically suspected in any of the patients; treating institutions detected the organism through matrix-assisted laser desorption/ionization–time-of-flight mass spectrometry (MALDI-TOF) testing of wound-derived coryneform isolates. MALDI-TOF is a rapid screening platform that uses mass spectrometry to identify bacterial pathogens. State public health laboratories confirmed *C. diphtheriae* through culture and sent isolates to CDC’s Pertussis and Diphtheria Laboratory for biotyping, polymerase chain reaction (PCR) testing, and toxin production testing. All isolates were identified as toxin-producing *C. diphtheriae*. The recommended public health response for cutaneous diphtheria is similar to that for respiratory diphtheria and includes treating the index patient with antibiotics, identifying close contacts and observing them for development of diphtheria, providing chemoprophylaxis to close contacts, testing patients and close contacts for *C. diphtheriae* carriage in the nose and throat, and providing diphtheria toxoid–containing vaccine to incompletely immunized patients and close contacts. This report summarizes the patient clinical information and response efforts conducted by the Minnesota, Washington, and New Mexico state health departments and CDC and emphasizes that health care providers should consider cutaneous diphtheria as a diagnosis in travelers with wound infections who have returned from countries with endemic diphtheria.

## Patient 1

In September 2015, a Minnesota woman aged 35 years returned from Somalia and sought medical care for a painful abdominal wound. *Staphylococcus aureus* and a coryneform isolate (identified as *C. diphtheriae* via MALDI-TOF and confirmed as toxin-producing) grew from the wound culture (Table). The patient was not tested for *C. diphtheriae* carriage. Throat and nasal swabs from four asymptomatic household contacts were obtained both before and at least 24 hours after a prophylactic course of penicillin; all cultures were negative for *C. diphtheriae*. The patient and household contacts were unimmunized but refused diphtheria toxoid–containing vaccines.

**TABLE:** Epidemiologic and clinical characteristics of four cases of toxin-producing cutaneous diphtheria — Minnesota, Washington, and New Mexico, 2015–2018

Characteristic	Patient 1	Patient 2	Patient 3	Patient 4
State of residence	Minnesota	Minnesota	Washington	New Mexico
Age (yrs)	35	48	12	42
Sex	F	M	F	M
Country of travel	Somalia	Ethiopia	Philippines	Philippines
DT-containing vaccination status	unvaccinated	unknown	UTD	unknown
Interval from onset of skin lesion to initial treatment	18 days	32 days	unknown	17 days
Wound culture findings	*Staphylococcus aureu*s, corynebacteria	Group A *Streptococcus, Pseudomonas*, corynebacteria	Corynebacteria	Group A *Streptococcus*, corynebacteria
*Corynebacterium diphtheriae* method of identification	MALDI-TOF	MALDI-TOF	MALDI-TOF	MALDI-TOF
*C. diphtheriae* biovar*	Mitis	Mitis	Mitis	Mitis
Treatment after *C. diphtheriae* identification	penicillin V	none; wound healed by time of identification	erythromycin	penicillin
No. of close contacts identified	4	0	16	3
DT-containing vaccination status of close contacts	4/4 unvaccinated	N/A	4/16 unvaccinated; 12/16 UTD	3/3 unvaccinated

**Abbreviations:** DT = diphtheria toxoid; F = female; MALDI-TOF = matrix-assisted laser desorption/ionization–time-of-flight mass spectrometry; M = male; N/A = not applicable; UTD = up-to-date.

* A biovar is a strain variant distinguishable by biochemical or physiologic characteristics.

## Patient 2

In September 2017, a Minnesota man aged 48 years returned from Ethiopia with an infected leg wound. The wound culture grew group A *Streptococcus, Pseudomonas*, and a coryneform isolate (identified as *C. diphtheriae* via MALDI-TOF and confirmed as toxin-producing). The patient was not tested for *C. diphtheriae* carriage, and a contact investigation was not undertaken because the patient lived alone and reported no close contacts. The patient reported that he had received a diphtheria toxoid–containing vaccine upon emigration to the United States 8 years earlier; therefore, no vaccine was administered. Because the wound had healed by the time the infecting organism was identified, no antibiotic treatment was administered.

## Patient 3

In September 2017, a Washington girl aged 12 years was evaluated for possible meningitis (which was unrelated to the cutaneous diphtheria later diagnosed) after travel to the Philippines. While she was receiving medical care, infected insect bites on her lower extremities were noted; wound cultures grew a coryneform isolate (identified as *C. diphtheriae* via MALDI-TOF and confirmed as toxin-producing). The patient was not tested for *C. diphtheriae* carriage. Sixteen household and other close contacts of the patient were identified. Nasal and throat swabs from 11 asymptomatic contacts were obtained before administration of a prophylactic course of erythromycin; all cultures were negative. Swabs were not collected from five contacts who had already started antibiotic prophylaxis. The patient and 12 contacts were up-to-date for diphtheria toxoid–containing vaccine and did not require additional doses. Four unvaccinated close contacts received diphtheria toxoid–containing vaccines.

## Patient 4

In February 2018, a New Mexico man aged 42 years returned from the Philippines with an exudative lower leg wound (Figure). Specimens were collected from the leg wound, and the culture grew group A *Streptococcus* and a coryneform isolate (identified as *C. diphtheriae* via MALDI-TOF and confirmed as toxin-producing). The patient was tested for *C. diphtheriae* carriage by nasal and throat swabs after antibiotics were administered, and both cultures were negative for *C. diphtheriae*. Nasal and throat swabs were collected from three asymptomatic household contacts before a prophylactic course of penicillin. All cultures were negative for *C. diphtheriae.* The patient’s vaccination status was unknown, and no contacts were up to date with their vaccinations; all received diphtheria toxoid–containing vaccines.

**FIGURE:**
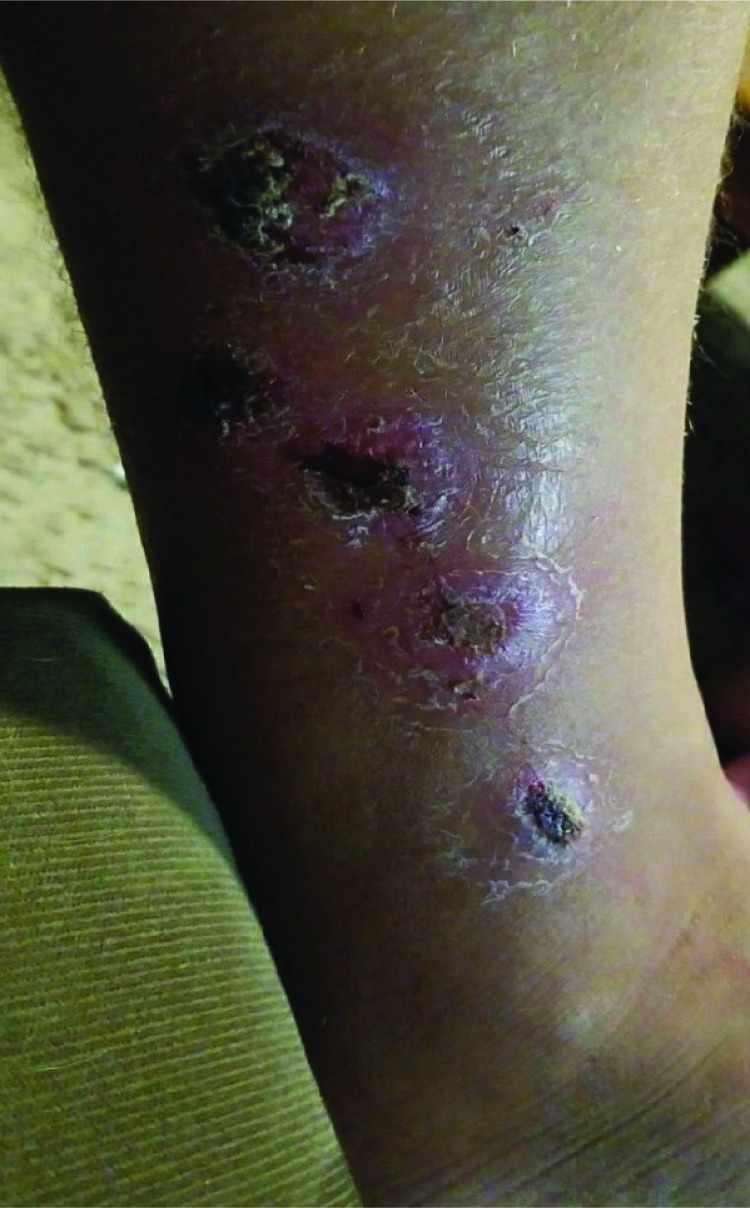
*Corynebacterium diphtheriae*–infected lower leg wound — New Mexico, 2018 Photo/New Mexico Department of Health (provided by patient 4 and used with permission)

## Discussion

Diphtheria is a rare, vaccine-preventable, bacterial disease caused by toxin-producing strains of *C. diphtheriae*. Infections are primarily respiratory or cutaneous and are transmitted from person to person by respiratory droplets or direct contact with discharge from skin lesions. Respiratory disease can be life-threatening and is characterized by the development of an adherent pseudomembrane in the upper respiratory tract. Cutaneous disease is typically characterized by well-demarcated ulcers that might have a membrane; the lesions are slow-healing and might act as a reservoir from which bacteria can be transmitted to susceptible contacts, potentially resulting in cutaneous or respiratory disease (*1*,*2*). Disease severity is mediated by successful bacterial expression of diphtheria toxin, encoded by a toxin gene introduced by corynebacteriophages. Nontoxin-producing strains of *C. diphtheriae* can also cause disease; it is generally less severe, although invasive disease associated with nontoxin-producing strains has been reported (*3*). Vaccination with diphtheria toxoid–containing vaccine might not prevent cutaneous colonization or infection with *C. diphtheriae* (*4*).

Respiratory diphtheria is nationally notifiable in the United States, but cutaneous diphtheria was not notifiable during 1980–2018; thus, the incidence of cutaneous diphtheria is not well defined (*4*). For reporting purposes, before 2019, a confirmed case of diphtheria was defined by clinically compatible respiratory disease and isolation of *C. diphtheriae*; confirmation of toxin production was not required.* However, to better identify disease with public health implications, a modification to the case definition was accepted by the Council of State and Territorial Epidemiologists and was implemented in January 2019.^†^ The modification restricts reporting to cases with toxin-producing disease, regardless of site.

Several common characteristics were observed among the patients with cutaneous diphtheria in this series, which might be useful for future case recognition. All had recently traveled to countries with endemic diphtheria; several European countries have also reported travel-related toxin-producing cutaneous diphtheria (*5*,*6*). *C. diphtheriae* was not clinically suspected in any of the patients and was only detected through laboratory testing. In three of four cases, *C. diphtheriae* was detected along with other more typical cutaneous pathogens, and similar coinfections have been described previously (*7*–*9*). To prevent delayed diagnosis and further disease transmission, it is important that health care providers be aware that diphtheria can manifest as a cutaneous infection, particularly in persons with wound infections and recent travel to countries with endemic diphtheria, even when *C. diphtheriae* is isolated with other potential pathogens.

When *C. diphtheriae* is identified through testing such as culture, PCR, or MALDI-TOF, it is critical that state and local public health laboratories submit specimens or isolates to CDC for confirmatory testing. CDC’s Pertussis and Diphtheria Laboratory routinely performs culture and biotyping to confirm *C. diphtheriae* and is currently the only laboratory in the United States that tests for toxin production. Based on available data from 1998 to 2017, 248 human *C. diphtheriae* isolates were tested at CDC’s Pertussis and Diphtheria Laboratory, including 130 (52%) cutaneous isolates. Among 243 isolates with known toxin production status, five (2%) were toxin-producing: three were cutaneous isolates (described in this report), and two were respiratory isolates from patients who did not have clinically compatible disease. The fourth cutaneous isolate described in this report was identified in 2018 and was outside the time frame of available data. Since 1998, both the number of isolates confirmed as *C. diphtheriae* by CDC’s Pertussis and Diphtheria Laboratory and the proportion of *C. diphtheriae* isolates originating from cutaneous sites have increased. During 1998–2011, an average of three isolates were confirmed as *C. diphtheriae* annually; this increased tenfold to 33 per year during 2012–2017 (CDC, unpublished data). Among the 130 cutaneous isolates, 95% were received during 2012–2017, possibly because of increased use of MALDI-TOF as a diagnostic tool. Current surveillance data might still underestimate the incidence of cutaneous diphtheria, because health care providers might not clinically suspect or test for diphtheria in patients, and because nonrespiratory diphtheria cases were not nationally notifiable during 1980–2018.

When suspected cases of *C. diphtheriae* are identified, state health departments should be notified to ensure that appropriate diagnostic testing (including culture and testing for toxin production) is completed and to facilitate prompt public health action. If an isolate is confirmed as toxin-producing diphtheria, public health interventions should be initiated. Treating patients with a 14-day course of erythromycin or penicillin to eradicate *C. diphtheriae* will reduce symptoms of infection and prevent transmission; treatment with diphtheria antitoxin is generally not recommended, unless signs of systemic toxicity are present. Close contacts of patients should be monitored for development of respiratory or cutaneous illness for 7–10 days after their last exposure. Close contacts include all household members, persons with a history of habitual, close contact with the patient, or persons directly exposed to patient secretions. For chemoprophylaxis, close contacts should receive a 7–10 day course of erythromycin or penicillin. Before antibiotic administration, diphtheria patients and their close contacts should have nasal and throat swabs collected for culture to test for *C. diphtheriae* carriage. Clearance of the organism should be confirmed after completion of the antibiotic course by repeat swabbing and testing. If repeat testing is still positive, another course of antibiotics should be administered. Finally, patients and close contacts who are not up-to-date with diphtheria vaccination should receive the recommended doses of diphtheria toxoid–containing vaccine (*4*).

The cases described in this report highlight the importance of recognizing cutaneous diphtheria in recent travelers to diphtheria-endemic countries with wound infections and the need for recommended diagnostic testing, including testing for toxin production, to implement a prompt public health response and prevent disease transmission.

**Summary**

**What is already known about this topic?**

Cutaneous diphtheria has not been notifiable in the United States since 1980, and U.S. disease incidence data are limited.

**What is added by this report?**

Toxin-producing *Corynebacterium diphtheriae* was identified in cutaneous wounds from four U.S. residents after return from international travel. Public health response for toxin-producing diphtheria includes treating patients, providing chemoprophylaxis to close contacts, testing patients and close contacts for *C. diphtheriae* carriage, and providing diphtheria toxoid–containing vaccine to incompletely immunized patients and close contacts.

**What are the implications for public health practice?**

Cutaneous toxin-producing diphtheria should be considered in travelers with wound infections who have returned from countries with endemic disease to permit prompt public health response and prevent disease transmission.

## Footnotes

* https://www.cste.org/resource/resmgr/PS/09-ID-05.pdf.

† https://www.cste.org/resource/resmgr/2018_position_statements/18-ID-03.pdf.
